# Practical Notes on Diagnosis and Treatment

**Published:** 1909-01-09

**Authors:** 


### Practical Notes on Diagnosis and Treatment.

**Addison's Disease.**

Good results have been reported in the treatment of a severe case of Addison's disease by (1) washing out the stomach with saline fluid, (2) restriction to fluid food supplemented by nutrient enemata, (3) the free administration of hydrochloric acid.

**Strontium Bromide in Epilepsy.**

I should be inclined to advise those who have epileptics under their care to give the bromide of strontium a fair trial, and I think they will find many cases in which this drug will have pre-eminence over the other bromide salts.—*Dr. J. M. Bennion.*

**Tumour of Spinal Cord.**

A patient with a tumour pressing on, say, the right side of his spinal cord would have paralysis of the right lower limb, with slight blurring of sensibility on that side, whereas in the left lower limb the defect of sensibility would be more pronounced, while the loss of motor power would be less so. This is known as Brown-Sequard's paralysis.—*Dr. Risien Russell.*

**Acute Pericarditis.**

In acute pericarditis opium is an invaluable remedy, for it allays the irritable excited action of the heart in a way no other drug does. No large amount is required. Small doses at frequent intervals, say five minims or so of laudanum every four hours, are all that is necessary. Many cases of rheumatic heart affections in the acute stage do better with opium than with any other drug.—*Dr. Samuel West.*

**Cancer of the Stomach.**

With a loss of weight which is continuous in spite of sufficient nourishment malignant disease should always be suspected.

**Intracranial Tumour.**

A patient suffering from intracranial tumour may first come under observation for an apoplectic seizure due to a large hæmorrhage having taken place into the growth.—*Dr. J. Mitchell Clarke.*

**Simple Goitre.**

Operations for innocent goître yield admirable results, and afford as a rule complete relief from all symptoms. The patient should be encouraged to be up and about within a very few days of the operation.—*Mr. James Berry.*

**Gall-Stones.**

We are often told that gall-stones may exist for years in the gall-bladder without causing symptoms. I do not believe for one moment that this statement is accurate. The truth is rather that those inaugural symptoms which are caused by the stones as they lie in the gall-bladder are not generally recognised.—*Mr. B. G. A. Moynihan.*

**Gastric Carcinoma.**

A peculiar somnolence is not infrequently noticed in those who are the subjects of gastric carcinoma, and this may attract the attention of the patient or his friends long before the diagnosis can be made with certainty. Look with grave suspicion on a doubtful history of dyspeptic symptoms in an individual over fifty who informs you that he cannot now do without an afternoon nap.

